# Torque teno virus: an improved indicator for viral pathogens in drinking waters

**DOI:** 10.1186/1743-422X-5-112

**Published:** 2008-10-03

**Authors:** Jennifer S Griffin, Jeanine D Plummer, Sharon C Long

**Affiliations:** 1 Department of Civil and Environmental Engineering, 100 Institute Road, Worcester Polytechnic Institute, Worcester, MA 01609, USA; 2 Department of Soil Science and Wisconsin State Laboratory of Hygiene, 2601 Agriculture Drive, Madison, WI 53718, USA

## Abstract

**Background:**

Currently applied indicator organism systems, such as coliforms, are not fully protective of public health from enteric viruses in water sources. Waterborne disease outbreaks have occurred in systems that tested negative for coliforms, and positive coliform results do not necessarily correlate with viral risk. It is widely recognized that bacterial indicators do not co-occur exclusively with infectious viruses, nor do they respond in the same manner to environmental or engineered stressors. Thus, a more appropriate indicator of health risks from infectious enteric viruses is needed.

**Presentation of the hypothesis:**

Torque teno virus is a small, non-enveloped DNA virus that likely exhibits similar transport characteristics to pathogenic enteric viruses. Torque teno virus is unique among enteric viral pathogens in that it appears to be ubiquitous in humans, elicits seemingly innocuous infections, and does not exhibit seasonal fluctuations or epidemic spikes. Torque teno virus is transmitted primarily via the fecal-oral route and can be assayed using rapid molecular techniques. We hypothesize that Torque teno virus is a more appropriate indicator of viral pathogens in drinking waters than currently used indicator systems based solely on bacteria.

**Testing the hypothesis:**

To test the hypothesis, a multi-phased research approach is needed. First, a reliable Torque teno virus assay must be developed. A rapid, sensitive, and specific PCR method using established nested primer sets would be most appropriate for routine monitoring of waters. Because PCR detects both infectious and inactivated virus, an *in vitro *method to assess infectivity also is needed. The density and occurrence of Torque teno virus in feces, wastewater, and source waters must be established to define spatial and temporal stability of this potential indicator. Finally, Torque teno virus behavior through drinking water treatment plants must be determined with co-assessment of traditional indicators and enteric viral pathogens to assess whether correlations exist.

**Implications of the hypothesis:**

If substantiated, Torque teno virus could provide a completely new, reliable, and efficient indicator system for viral pathogen risk. This indicator would have broad application to drinking water utilities, watershed managers, and protection agencies and would provide a better means to assess viral risk and protect public health.

## Background

The connection between fecal contamination of drinking water and outbreaks of disease from waterborne pathogens has been established for more than a century [1]. Because it would not be feasible to monitor directly for every known pathogen, indicator organisms, which correlate with fecal contamination and suggest health risk, are used instead [2,3]. In water supply systems, monitoring for total coliforms, fecal coliforms, and *E. coli *is regulated under the Total Coliform Rule (TCR) [4]. However, these bacterial indicators are not always 100% protective of public health, particularly from enteric viruses. Waterborne disease outbreaks of viral etiology have occurred in systems in which coliforms were absent, and instances of coliform presence in violation of the TCR are not always associated with adverse public health outcomes [5-7].

The use of coliforms as indicators of viral pathogen risk is problematic for several reasons:

1) There is a lack of association between coliforms and human enteric viruses in the environment. Bacterial indicators have low predictive ability for enteric viruses [8,9] and low or no correlation to viruses [10-16].

2) The fate of coliforms and viral pathogens in environmental systems is disparate. Coliform bacteria are more susceptible than enteric viruses to extremes in pH, salinity, and temperature [9,17-19]. In addition, bacteria are more easily removed by filtration through natural aquifer systems [13,20-22]. Overall, virus persistence and mobility generally exceed that of bacteria in environmental waters [9,23].

3) Coliforms and viral pathogens have distinct resistance patterns in engineered treatment processes [24]24, and infectious viruses have been found in finished waters that are coliform negative [25,26]. Physical removal of viruses through treatment systems, for instance by ultrafiltration or microfiltration membranes, is more challenging than removal of bacteria [27-32]. In addition, many enteric viruses are more resistant than bacteria to disinfection with chlorine and ultraviolet radiation [8,33-36].

Several alternatives to bacterial indicators have been proposed. Coliphages exhibit similarities to enteric viruses regarding environmental transport and survival [37,38]. However, coliphage survival characteristics vary by season [39] and by coliphage group [12,40-43]. In addition, coliphages may continue to replicate in surviving bacterial hosts after being shed in feces, thus exhibiting much greater persistence than human enteric viruses in receiving waters [9,44]. Alternatively, only a small percentage of human or animal fecal samples test positive for coliphages [45,46] so these viruses may be too sparse to detect in some environmental waters.

Some researchers have suggested enteroviruses or noroviruses as indicators of other enteric viruses [47,48]. However, these viruses exhibit seasonal fluctuations and epidemic spikes [16,49]. In addition, quantification of infectious noroviruses *in vitro *has only recently been accomplished using 3-D cell culture [50], which is well beyond the analytical capabilities of typical water testing laboratories. Adenovirus has been proposed as an indicator because of its remarkable resistance characteristics and lack of seasonal variability. However, this virus did not correlate with hepatitis A virus or enteroviruses in urban waterways [51].

We hypothesize that Torque teno virus (TTV) is a superior indicator of enteric viruses compared to traditional bacterial indicators and proposed viral indicators. TTV is an enterically transmitted human virus, but it exhibits characteristics that distinguish it from other enteric viruses. Recent studies toward understanding the biology and occurrence of TTV provide preliminary support for our hypothesis.

## Presentation of the hypothesis

TTV is a recently discovered non-enveloped virus with a single-stranded, circular DNA genome [52-54]. TTV isolates are remarkably variable with 47–70% divergence at the amino acid level [55,56]. TTV divergence is unevenly distributed across the genome; hypervariable regions exist within the coding region [57], and the untranslated region contains conserved regulatory sequences [58].

Initially, TTV was described as a novel hepatitis virus [52], but it was later determined that TTV circulates in a large proportion of healthy individuals [59-61] with an average worldwide prevalence estimated at 80% [62,63]. The virus appears to elicit both persistent and transient infections [52]. Transmission of TTV is primarily by the fecal-oral route [63], but it is detected in a variety of human tissues and fluids, including plasma and serum [64-68]. Many attempts have been made to assign a pathology to TTV, but none have been substantiated. In fact, Griffiths [69] and Simmonds *et al. *[70] have suggested that TTV may constitute the first known commensal human virus.

A few investigators have tracked TTV in the environment or in treatment systems. Their results suggest that TTV may co-locate with various enteric viruses. Currently, little is known about the environmental stability of TTV, although Takayama *et al*. [71] demonstrated that TTV infectivity was not lost after 95 hours of dry heat treatment. Investigators suspect that TTV particles are highly resistant to environmental stressors [72].

In polluted streams of Brazil, TTV was found to be spatially and temporally constant [61], and the TTV positivity rate of 92.3% paralleled the positivity rate reported by de Paula *et al*. [73] for hepatitis A virus in the same geographic region. In Italy, river water samples receiving waste treatment effluent were found to contain TTV and other enteric viruses [72]. TTV and rotavirus occurred either simultaneously or within 1 month's sampling period of each other. In addition, TTV occurred 1–2 months after enterovirus was detected and simultaneously or within 2 months of noroviruses g1 and g2 in all but one case.

Vaidya *et al*. [59] compared sewage treatment plant influent and effluent concentrations of TTV and hepatitis A and E viruses via PCR and observed that raw sewage prevalence of TTV DNA was statistically similar to the prevalence of hepatitis E virus RNA and hepatitis A virus RNA. Following treatment, hepatitis A virus RNA was significantly reduced, but the reductions in TTV and hepatitis E virus genetic material were not statistically significant. When TTV was monitored through activated sludge wastewater treatment plants in Japan, researchers reported that the TTV genome was detected with 97% frequency in influent, 18% in secondary effluent after activated sludge but before chlorination, 24% in final effluent after chlorination, and 0% in effluent for reuse following filtration and ozonation [60]. In contrast, coliforms decreased sequentially with each step in the treatment process, and the concentration of coliforms did not correlate with the number of positive TTV samples collected at any step.

As a putative indicator, TTV should be abundant where water is not adequately treated and diarrheal disease is common and should exist at low or undetectable levels where water treatment leads to clean, potable water. Poor sanitation may increase TTV transmission by the fecal-oral route, as the countries of Bolivia and Burma – both with high risks of waterborne disease – have TTV incidences of 82% and 96%, respectively, among otherwise healthy individuals [74]. In contrast, TTV prevalence in the United States is estimated to be 10% [75]. It is hypothesized that at this prevalence, TTV would be present in most environmental samples at levels high enough to be detected using PCR [63] with the exception of contamination resulting from single septic systems.

## Testing the hypothesis

A three-phased plan of research is necessary to determine the value of TTV as an indicator for viral pathogens.

### Phase I – Develop reliable TTV assay

PCR indicates the presence/absence of a target sequence and would yield a positive result for a non-infectious viral particle if the particle's genetic material was intact. The presence of viral nucleic acid at a site nevertheless indicates that contamination occurred in the recent past and suggests that the site is susceptible to future contamination [76]. The rapid nature of PCR makes it an ideal tool for periodic monitoring of water sources.

Because viruses are present in low concentrations in environmental waters, it is necessary to concentrate water samples by several orders of magnitude prior to PCR analysis. However, sample concentration also may concentrate inhibitors of DNA polymerase. The use of hollow fiber ultrafiltration is proposed. This method is effective for concentrating MS2 coliphage, noroviruses, and adenoviruses for subsequent enumeration or PCR detection [[77]; Sibley SD, personal communication]. The selection of primers against conserved regions of the TTV genome is crucial for accurately detecting all TTV isolates. In addition to amplifying a conserved sequence, nested or seminested PCR is proposed; this technique approaches a resolution of one TTV genome/sample [53,62,78].

If TTV is to be used as an indicator – particularly in a treatment system in which viral particles may be inactivated but not removed – a method must be available to determine TTV infectivity. *In vitro *infection by TTV has been demonstrated in activated peripheral blood mononuclear cells and the Chang liver cell line [79-81]. Either of these may be candidates for infectivity assessment. Chang liver cells exhibit cytopathic effects 2–3 days after inoculation with TTV [81] so this cell line may be useful for rapid identification of infectivity.

### Phase II – Monitor TTV in sources

In order to determine the utility of TTV as an indicator, the occurrence, density, and persistence of TTV in feces, wastewater, and environmental waters need to be evaluated. Geographically diverse samples should be collected during all seasons to assess both spatial and temporal stability. The persistence of the TTV genome has not been described in environmental waters, but researchers have reported that TTV DNA from fecal extracts degrades by approximately 3 log_10 _within 1 week when monitored by real-time PCR at 37°C [81]. Once these data are gathered, the results can be compared to coliforms, coliphages, and total culturable viruses to determine whether TTV co-locates with other enteric viruses and/or other indicators.

### Phase III – Monitor TTV through drinking water treatment

The fate of TTV through drinking water treatment processes needs to be assessed. Prior research has demonstrated removal/inactivation of TTV through wastewater treatment [60], but data are lacking for municipal drinking waters. As with source monitoring, spatial and temporal diversity of the sampling protocol is necessary. Co-monitoring coliforms, coliphages, and total culturable viruses should be performed to demonstrate the relative resistance of TTV to treatment effects and to determine relationships, if any, among TTV, enteric viruses, and indicators.

## Implications of the hypothesis

Because of the shortcomings of traditional bacterial indicator organisms to accurately indicate viral risk, novel indicator or monitoring systems are needed. If the indicator potential of TTV is substantiated, a TTV indicator system could complement or replace traditional bacterial indicators for the detection of human enteric viruses in environmental samples. The ability to assess viral pathogen risk would be enhanced, and ultimately, public health would be better protected.

## Competing interests

The authors declare that they have no competing interests.

## Authors' contributions

All authors contributed equally to this manuscript. All authors read and approved the final manuscript.

## Authors' information

JSG is a graduate student at WPI with expertise in molecular, biochemical, and virologic techniques. JSG is well versed in PCR, including real-time and endpoint PCR. Her technical skills include mammalian, yeast, and bacterial cell culture; genetic engineering; viral protein biochemistry; and basic viral infection, propagation, and storage techniques. JDP is a faculty member in Environmental Engineering with 15 years experience in source water protection, microbial source tracking, and physical/chemical water treatment. SCL is a faculty member in Soil Science and Director of Microbiology at a State Hygiene Laboratory. She has over 20 years of expertise in watershed management, water quality analysis, indicator organism microbiology and public health issues.
